# A cell-based high-content screen identifies isocotoin as a small molecule inhibitor of the meiosis-specific MEIOB–SPATA22 complex^†^

**DOI:** 10.1093/biolre/ioaa062

**Published:** 2020-04-25

**Authors:** Yang Xu, Rong Liu, N Adrian Leu, Lei Zhang, Ilsiya Ibragmova, David C Schultz, P Jeremy Wang

**Affiliations:** 1 Department of Biomedical Sciences, University of Pennsylvania School of Veterinary Medicine, Philadelphia, Pennsylvania, USA; 2 School of Basic Medical Sciences, Wuhan University, Wuhan, Hubei Province, China; 3 High-Throughput Screening Core, University of Pennsylvania, Philadelphia, Pennsylvania, USA

**Keywords:** meiosis, MEIOB, SPATA22, isocotoin, male contraception

## Abstract

MEIOB and SPATA22 are meiosis-specific proteins, interact with each other, and are essential for meiotic recombination and fertility. Aspartic acid 383 (D383) in MEIOB is critical for its interaction with SPATA22 in biochemical studies. Here we report that genetic studies validate the requirement of D383 for the function of MEIOB in mice. The *Meiob*^D383A/D383A^ mice display meiotic arrest due to depletion of both MEIOB and SPATA22 proteins in the testes. We developed a cell-based bimolecular fluorescence complementation (BiFC) assay, in which MEIOB and SPATA22 are fused to split YFP moieties and their co-expression in cultured cells leads to the MEIOB–SPATA22 dimerization and reconstitution of the fluorophore. As expected, the interaction-disrupting D383A substitution results in the absence of YFP fluorescence in the BiFC assay. A high-throughput screen of small molecule libraries identified candidate hit compounds at a rate of 0.7%. Isocotoin, a hit compound from the natural product library, inhibits the MEIOB–SPATA22 interaction and promotes their degradation in HEK293 cells in a dose-dependent manner. Therefore, the BiFC assay can be employed to screen for small molecule inhibitors that disrupt protein–protein interactions or promote degradation of meiosis-specific proteins.

## Teaser

A cell-based bimolecular fluorescence complementation screening assay identifies isocotoin that promotes degradation of MEIOB and SPATA22.

## Introduction

Male reproduction is a multistep process involving hormonal regulation, spermatogenesis, and sperm maturation [1]. In theory, each step is a potential site for disruption of male fertility, but some steps may be more amenable to intervention than others. To date, the development of hormonal male contraception has focused on inhibition of spermatogenesis by suppression of the hypothalamic–pituitary secretion of gonadotropins following testosterone replacement therapy [2, 3]. In contrast, nonhormonal male contraceptive lead compounds target Sertoli cells and germ cells [4–6]. Ideally, acceptable nonhormonal male contraceptives should be effective and reversible with minimal side effects. To date, no such male contraceptives are available. This is somewhat surprising, because spermatogenesis appears highly susceptible to perturbations and at least 4% of all genes are specifically involved in male reproduction [7]. In particular, genetic studies in mice have identified more than 600 genes that are specifically or preferentially involved in the regulation of fertility, providing a plethora of potential molecular targets for contraception in humans [8–10].

Most mouse genetic mutations affecting fertility have been characterized following targeted disruption in embryonic stem cells, forward genetic mutagenesis screens, and CRISPR/Cas9-mediated genome editing. Studies of these mouse mutants have defined a number of fundamental biological processes in germ cell development, gamete maturation, and fertilization [10, 11]. While many of these mutants exhibit pleiotropic defects, a fraction of them give rise to “pure sterile” phenotypes with no observed somatic defects. Such “pure sterile” mutants potentially identify gene targets for developing specific male contraceptives with minimal side effects. Meiotic recombination, an essential process for spermatogenesis, could be targeted for male contraception, since most protein components of meiotic recombination are specifically expressed in germ cells.

MEIOB (meiosis-specific with oligonucleotide-binding motif) is a meiosis-specific single-stranded DNA-binding protein. We identified MEIOB in a genome-wide proteomics screen for meiotic chromatin-associated proteins in mice [12]. SPATA22 (spermatogenesis associated 22) is also a meiosis-specific protein essential for meiotic recombination [13–15]. Both *Meiob* and *Spata22* are specifically expressed in meiotic germ cells but not in somatic tissues. MEIOB and SPATA22 co-localize as foci on meiotic chromosomes [12]. Both *Meiob-* and *Spata22*-deficient mice are viable and healthy. However, inactivation of either *Meiob* or *Spata22* causes meiotic failure in both males and females, thus resulting in “pure sterility” in mice [12, 16]. Mutations in the human *MEIOB* gene cause infertility in men and primary ovarian insufficiency in women [17–19]. In mouse testes, MEIOB interacts with SPATA22. In addition, chromatin localization of MEIOB and SPATA22 is mutually dependent [12]. Based on these findings, blockade of MEIOB–SPATA22 interaction by chemical inhibitors could disrupt spermatogenesis, thus providing a novel male contraceptive target.

MEIOB exhibits homology (23% aa identity) with replication protein A1 (RPA1) in the OB (oligonucleotide-binding) domain and thus is a meiosis-specific paralogue of RPA1 [12]. MEIOB forms a complex with SPATA22 and RPA [20]. RPA, a heterotrimer of RPA1, RPA2, and RPA3, is a ubiquitously expressed ssDNA-binding complex, which is required for DNA replication, DNA repair, and meiotic recombination [21–23]. Biochemical studies have shown that MEIOB and SPATA22 form an obligate complex through defined interaction domains [20]. The SPATA22-binding domain (aa 294–450) in MEIOB is distinct from its ssDNA/RPA1-binding domain. The aspartic residue D383 in MEIOB is required for its interaction with SPATA22, as mutation of D383 to an alanine completely abolishes the MEIOB–SPATA22 interaction in cultured cells [20]. The MEIOB-SPATA22 complex interacts with the RPA heterotrimeric complex, but their localizations to DNA double-strand breaks are independent [12, 23].

Enzymes such as kinases have a defined docking pocket for their substrates and thus are traditionally favorite targets for drug development. In contrast, protein–protein interactions have not been traditionally targeted for therapeutic purposes, partly because of the low success in finding small molecules that can inhibit or disrupt the relatively large interfaces often involved in protein–protein interactions. However, despite these relatively large binding interfaces, the majority of binding energy usually involves only a few key residues within an interaction “hot spot” [24]. Protein–protein interactions have been targeted for drug development [25]. Notably, inhibitors that block protein interactions have been identified for chromatin-binding proteins and apoptosis proteins such as p53 and Bcl-2 [26]. In addition, a small molecule, JQ1, blocks the binding of BRDT to acetylated histone, resulting in reversible contraceptive effects in mice [5]. Given the largely untapped vast pharmacological space of the protein interactome, protein–protein interactions have become increasingly viable targets for drug development. Here we demonstrate that the D383 residue in MEIOB is essential for its interaction with SPATA22 in the testes. To screen for potential small molecule inhibitors of the MEIOB–SPATA22 interaction, we developed a bimolecular fluorescence complementation (BiFC) assay [27–29]. High-content screening of compound libraries identified isocotoin as a hit compound for degradation of MEIOB and SPATA22.

## Materials and methods

### Ethics statement

Mice were monitored daily in a barrier vivarium and under veterinarian care from University Laboratory Animal Resources at the University of Pennsylvania. All animal experimental procedures were approved by the Institutional Animal Care and Use Committee at the University of Pennsylvania.

### Generation of *Meiob*^D383A^ mice

The guide RNA (GTTGATCTTACTGACCACAC) was cloned into the px330 vector (Addgene) and transcribed in vitro (Supplementary Figure S1A) [30]. The ssDNA repair template contains the D383A mutation (GAC to GCG): 5’-GAAGCCTCCAATACTTGTACAATTTGCAACCAAGATTCTTCCAGATTGAAATCTTTTTTTCTCAGTTTTGATGTGCTAGTTGATCTTACTGCGCACACTGGAACCCTCCATTCCTGCAGTCTCTCAGGAAGTATTGCTGAGGAAACTTTGGGCTGCACGGTAAATAGCAGAAAGGAAAAAATAG-3′. A 50 μl mixture of Cas9 mRNA (catalogue number L-7206, TriLink, 100 ng/μl), sgRNA (50 ng/μl), and ssDNA template (100 ng/μl) was used for microinjection of ~ 60 zygotes (1–2 picoliter per zygote). All zygotes were transferred into three recipient females. Twenty-two founder mice were obtained. The *Meiob*^D383A^ allele was genotyped by PCR (205 bp) with primers 5’-GATGTGCTAGTTGATCTTACTGCG-3′ and 5’-GATGGCTTTTCCTAAGATCGCTTC-3′. The wild-type allele was genotyped by PCR (205 bp) with primers 5’-GATGTGCTAGTTGATCTTACTGAC-3′ and 5’-GATGGCTTTTCCTAAGATCGCTTC-3′. The genomic DNA around the D383A mutation was amplified by PCR (655 bp) with primers 5’-GTGCGTCTATGGTCTAGATTTGTG-3′ and 5’-GGCAGCTCATAACCATCTTTAACT-3′, subcloned and sequenced. Sanger sequencing revealed frame-shift (FS) mutations in the apparently “wild-type” alleles (Supplementary Figure S1B and C). We obtained the following founders: *Meiob*^D383A/FS^, *Meiob*^D383A/D383A^, and *Meiob*^FS/FS^. Four *Meiob*^D383A/FS^ males were bred with wild-type females, but none of them produced progenies.

### Generation of MEIOB–SPATA22 BiFC constructs

To generate the NYFP-MEIOB WT construct, NYFP (consisting of amino acids 1–173 of mVenus) (Addgene) was cloned into the HindIII/KpnI sites of the pcDNA3.1/myc-His B plasmid vector (Invitrogen), and mouse *Meiob* cDNA was cloned into the BamHI/EcoRI sites. The NYFP-MEIOB D383A construct was identical to the NYFP-MEIOB WT construct except for the point mutation D383A in MEIOB. To generate the CYFP-SPATA22 construct, CYFP (consisting of amino acids 155–239 of mVenus) was cloned into the HindIII/KpnI sites of the pcDNA3.1/myc-His B plasmid vector, and mouse *Spata22* cDNA was cloned into the BamHI/XhoI sites.

The BiFC WT construct consisted of NYFP-MEIOB WT, CYFP-SPATA22, and mCherry and was generated based on the NYFP-MEIOB WT construct. CYFP-SPATA22 was subcloned into the EcoRI/XbaI sites of the NYFP-MEIOB WT construct. mCherry was subcloned into the XbaI/ApaI sites. NYFP-MEIOB and CYFP-SPATA22 were separated by E2A peptide (GSGQCTNYALLKLAGDVESNPGP) inserted at the EcoRI site. CYFP-SPATA22 and mCherry were separated by F2A peptide (GSGVKQTLNFDLLKLAGDVESNPGP) inserted at the XbaI site. The BiFC D383A construct was identical to the BiFC WT construct except for the point mutation D383A in MEIOB. All the constructs were verified by Sanger sequencing on an ABI 3730 DNA analyzer.

### Establishment of Tet-inducible BiFC stable cell lines

The Flp-In T-REx-293 cell line was purchased from Thermo Fisher (catalogue number R78007) and maintained in DMEM/high glucose (MediaTech) supplemented with 10% FBS (Sigma), 1× penicillin–streptomycin (Invitrogen), 100 μg/ml zeocin, and 15 μg/ml blasticidin. The pcDNA5-BiFC construct was generated by subcloning the BiFC cassette containing NYFP-MEIOB-E2A-CYFP-SPATA22-F2A-mCherry into the NotI/XhoI sites of pcDNA5/FRT/TO-neo (catalogue number 41000, Addgene). To generate the BiFC MEIOB WT stable cell line, parental Flp-In T-Rex-293 cells were transfected with pPGKFLPobpA (catalogue number 13793, Addgene) and pcDNA5-BiFC at a 9:1 ratio. Twenty-four hours later, stable cell lines were selected under 400 μg/ml G418 and 15 μg/ml blasticidin for 10 days. A total of 15 individual clones were recovered in a 96-well plate through colony picking, and BiFC WT Clone No. 20 was saved for all experiments. This cell line (No. 20) was maintained in DMEM/high glucose supplemented with 10% FBS, 1× penicillin–streptomycin, 200 μg/ml G418, and 15 μg/ml blasticidin.

Likewise, we established Nef-NYFP-E2A-Nef-CYFP-F2A-mRFP BiFC tetracycline-inducible stable cell line (Clone No. 2) using the previously reported construct as a template [29]. Nef forms a homodimer [29].

### Compound libraries

We screened a total of 47 800 compounds, including 800 compounds from MicroSource natural product library, 3000 bioactive compounds from SelleckChem, 11 000 compounds from ChemDiv’s SMART library, and 33 000 compounds from ChemBridge’s Core set. Compounds were suspended in DMSO, arrayed in columns 3–22 of 384-well microplates, and stored at −20 °C. Library plates were thawed a maximum of 10× to maintain compound integrity.

### High-throughput screening

We seeded 4000 HEK293:FLP-InT-REx-BiFC#20 cells in a volume of 20 μl per well of 384-well clear-bottom, black-walled microplate (catalogue number 3764, Corning) precoated with 25 μg/ml collagen I using a Multidrop™ Combi Reagent Dispenser (Thermo Scientific). Cells were allowed to attach overnight at 37 °C, 5% CO_2_ in a humidified chamber. Compounds (50 nl) were transferred to assay plates using a 384, 50 nl slotted pin tool (V&P Scientific) and a JANUS Automated Workstation (Perkin Elmer). Compounds/drugs were added to a final concentration of 20 μM in 0.2% DMSO. We dispensed 5 μl of 10 μg/ml tetracycline diluted in growth medium to columns 1–23 using a Multidrop™ Combi Reagent Dispenser (Thermo Scientific) for a final concentration of 2 μg/ml. Column 24 received 5 μl growth medium. Cells were incubated at 37 °C, 5% CO_2_ for 24 h, and then fixed with 4% formaldehyde. Nuclei were stained with 4 μg/ml Hoechst 33342 dye (Sigma). Cells were imaged at 10× on an automated ImageXpress Micro (Molecular Devices, Sunnyvale CA).

### Data analysis

The total number cells, YFP+, RFP+, double positive, YFP integrated intensity, and RFP (mCherry) integrated intensity were quantified using a multiwavelength cell scoring algorithm in MetaXpress 5.3.0.5 (Molecular Devices, Sunnyvale, CA). For the pilot screening of the MicroSource and SelleckChem libraries, raw values from tetracycline-induced and non-induced wells were aggregated and used to calculate z’-factors for each assay plate, as a measure of assay performance and data quality. The total number of cells, YFP+, RFP+, double positive cells (YFP + RFP+), YFP integrated Intensity, RFP Integrated Intensity, and YFP/RFP intensity ratio were normalized to aggregated negative plate control wells (i.e., DMSO) and expressed as percentage of control [POC = ((Test well)/(DMSOavg)) × 100] and z-score [Z = (DMSOavg-Test well)/(DMSOsd)], a measure of standard deviations away from the mean of the aggregated negative controls, in Spotfire (PerkinElmer). Candidate hits were defined as compounds that inhibited YFP intensity by three standard deviations from the mean without affecting total number of cells and RFP intensity.

For the data analysis of screening of ChemDiv’s SMART library and ChemBridge’s core set, raw values from tetracycline-induced wells with DMSO (negative control) and isocotoin (positive control) were aggregated and used to calculate z’-factors for each assay plate, as a measure of assay performance and data quality. Candidate hits were defined by the following criteria: (1) YFP/RFP z-score ≥ −3; total cell POC > 80% (filter for cell toxicity); RFP integrated intensity −3 < z-score < 3 (secondary filter for toxicity and autofluorescence).

### Compound synthesis, induction of protein expression in stable cell lines, co-immunoprecipitation, and Western blot analysis

200 mg of isocotoin (C_14_H_12_O_4_; CAS# 81525-12-4; MW, 244) was synthesized at the Chemical and Nanoparticle Synthesis Core at the University of Pennsylvania. Isocotoin was dissolved in DMSO, aliquoted, and stored in lightproof tubes at −20 °C. VER-49009 was purchased from SelleckChem. The BiFC stable cell line #20 was used in all experiments. Expression of NYFP-MEIOB and CYFP-SPATA22 was induced by adding tetracycline (1 μg/ml) in the culture media. Cells were harvested for fluorescence imaging. YFP was imaged under the FITC channel, and mCherry was imaged under the Texas Red channel. Cells were lysed using the lysis buffer [62.5 mM Tris-HCl (pH 6.8), 3% SDS, 10% glycerol, and 5% 2-mercaptoethanol] for Western blotting analysis. Epitope-tagged MEIOB and SPATA22 expression constructs, transfection, and co-immunoprecipitation (Figure 3D) were previously described [20]. The following primary antibodies were used: rabbit anti-MEIOB antibody (1:250) [12], rabbit anti-SPATA22 (1:200, catalogue number 16989-1-AP, ProteinTech Group), anti-RPA1 (1:50, catalogue number Ab87272, Abcam), anti-RPA2 (1:50, UP2436, custom-made) [23], anti-V5 monoclonal (1:4000, catalogue number R960-25, Invitrogen), anti-FLAG monoclonal (1:10000, catalogue number F1804, Sigma), rabbit anti-GFP (1:1000, catalogue number ab290, Abcam), and mouse anti-ACTB monoclonal antibody (1:10000, catalogue number A5441, clone AC-15, Sigma). YFP fluorescence, mCherry fluorescence, and Western blot band quantifications were performed using ImageJ (https://imagej.nih.gov/ij/). IC50 was calculated using GraphPad Prism 8.3.0 (GraphPad software, LLC). All quantification experiments were performed at least three times.

## Results

### The aspartic acid D383 in MEIOB is required in vivo

**Figure 1:**
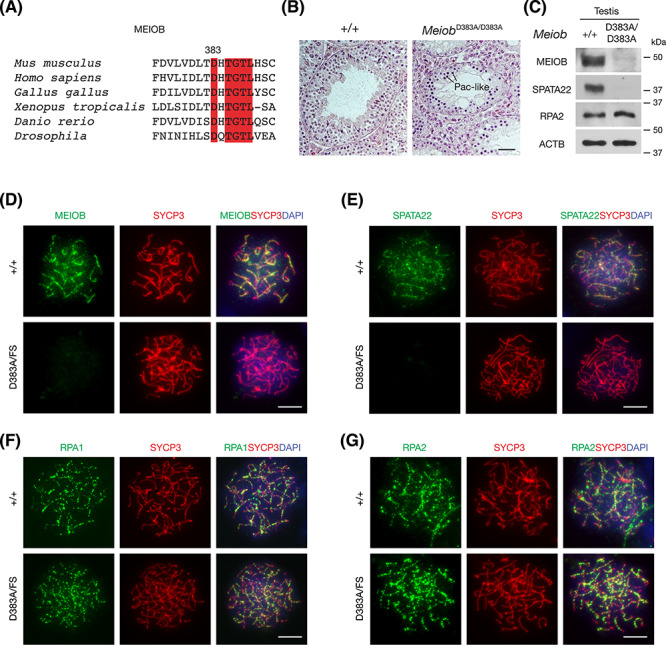
The MEIOB D383A mutation causes meiotic arrest and infertility in mouse. (A) Conservation of the D383 residue in MEIOB across species. The conserved residues are highlighted in red. (B) Histological analysis of testes from 8-week-old wild-type and *Meiob*^D383A/D383A^ mice. Pac-like, pachytene-like. Scale bar, 50 μm. (C) Western blot analysis of wild-type and *Meiob*^D383A/D383A^ testes. ACTB serves as a loading control. (D–G) Immunofluorescence analysis of surface nuclear spreads of spermatocytes from adult wild-type and *Meiob*^D383A/FS^ (founder tag# 74). Spread nuclei of spermatocytes were immunostained with anti-MEIOB (D), anti-SPATA22 (E), anti-RPA1 (F), and anti-RPA2 (G). DNA was stained with DAPI. Scale bars, 10 μm.

Our previous biochemical studies identified D383 as a critical residue for MEIOB–SPATA22 interaction [20]. The D383 residue is evolutionarily conserved in diverse species from *Drosophila* to human (Figure 1A). The D383A substitution in MEIOB abolished its interaction with SPATA22 when expressed in HEK293 cells [20]. In the current study, we sought to test the functional consequence of this substitution *in vivo*. To generate the *Meiob*^D383A^ allele, a guide RNA flanking *Meiob* codon 383 and an ssDNA template containing D383A mutation (GAC—GCG) were designed for CRISPR/Cas9-mediated genome editing in mouse zygotes (Supplementary Figure S1A). Out of 22 founder mice, a total of 15 *Meiob*^D383A/FS^ (9 males and 6 females; FS, frame shift) and 4 *Meiob*^D383A/D383A^ (1 male and 3 females) mice were obtained. No *Meiob*^D383A/+^ mice were produced due to frame-shift nonsense mutations in the apparent “+” allele. No germline transmission of the *Meiob*^D383A^ point mutation was obtained due to the infertility of both *Meiob*^D383A/FS^ and *Meiob*^D383A/D383A^ mice in both sexes. However, we could not exclude the remote possibility that off-target mutations might contribute to the infertility phenotypes.

All the *Meiob* mutant males exhibited sharply reduced testis size and meiotic arrest (Figure 1 and Supplementary Figure S1). In the *Meiob*^D383A/D383A^ testis, the most advanced spermatocytes were pachytene-like (Figure 1B). Western blot analysis revealed that MEIOB and SPATA22 were absent in the *Meiob*^D383A/D383A^ testis. The *Meiob*^D383A/FS^ testes were also significantly smaller than the wild-type testes (Supplementary Figure S1B and C) and displayed meiotic arrest (Supplementary Figure S1D). The frame-shift mutations led to the C-terminal truncation of the SPATA22-binding domain (aa 294–450) of MEIOB and thus were expected to disrupt the interaction (Supplementary Figure S1B and C) [20]. MEIOB and SPATA22 formed foci on meiotic chromosomes in wild-type spermatocytes but not in *Meiob*^D383A/FS^ spermatocytes (Figure 1D and E). RPA interacts and colocalizes with MEIOB/SPATA22 in spermatocytes [12]. In contrast, RPA2, a subunit of RPA, was abundantly expressed in the *Meiob*^D383A/D383A^ testis (Figure 1C). Like in wild-type spermatocytes, RPA1 and RPA2 foci were abundant in *Meiob*^D383A/FS^ spermatocytes, showing that RPA localization is independent of MEIOB (Figure 1F and G). Therefore, *Meiob*^D383A/D383A^ and *Meiob*^D383A/FS^ males displayed the same meiotic arrest phenotype as *Meiob*^−/−^ males [12]. Taken together, our study of the point mutant mice demonstrates that the interaction-disrupting mutation (D383A) leads to degradation of MEIOB (D383A) and SPATA22 in testes.

### Development of a BiFC assay for MEIOB–SPATA22 interaction

**Figure 2:**
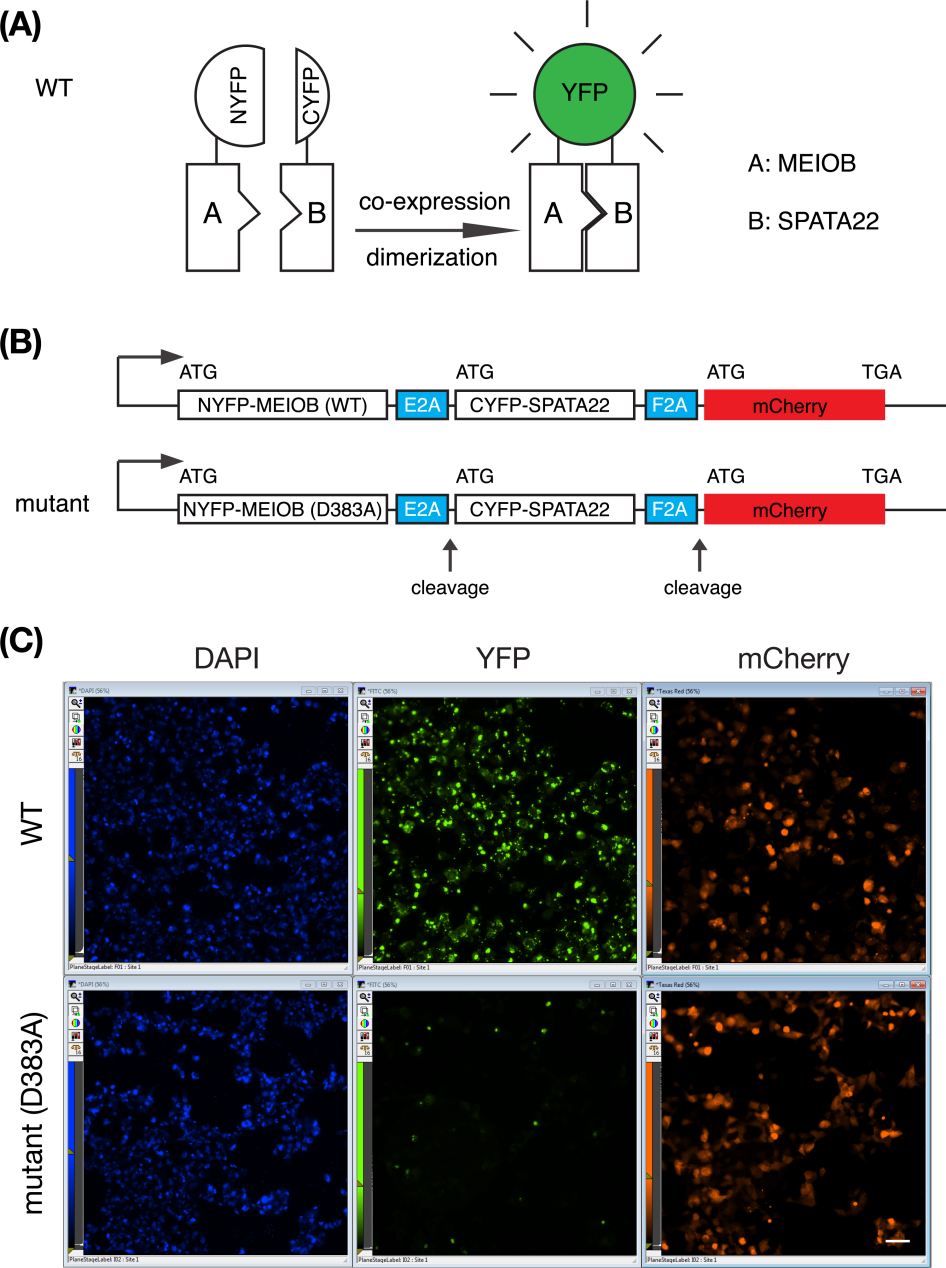
A cell-based high-content screening assay for MEIOB–SPATA22 interaction. (A) Bimolecular fluorescence complementation assay for protein–protein interactions. Binding of proteins A (MEIOB) and B (SPATA22) enables reconstitution of the fluorophore core of YFP. (B) Schematic design of a multi-protein tricistronic expression construct for the BiFC assay. Two self-cleaving 2A peptide sequences are from picornavirus. E2A: equine rhinitis A virus. F2A: foot-and-mouth disease virus. (C) Fluorescence readout of the BiFC assay. HEK293T cells were transfected with the wild-type construct or the mutant construct in the 384-well format. Cells were imaged 48 h post transfection. Only one well is shown per construct. Scale bar, 50 μm.

The bimolecular fluorescence complementation (BiFC) assay is a cell-based fluorescence technology for characterizing protein–protein interactions [28]. Yellow fluorescent protein (YFP) is partitioned into two parts: NYFP and CYFP. These two fragments are not fluorescent when separate but reconstitute the fluorophore and become fluorescent when brought into close proximity (Figure 2A). To develop a cell-based BiFC assay for protein–protein interactions, we constructed a tricistronic multi-protein expression vector (Figure 2B). In this construct, three proteins were expressed: NYFP-MEIOB (wild type), CYFP-SPATA22, and red fluorescent protein monomeric Cherry (mCherry). A “self-cleaving” 2A peptide was inserted between the fusion proteins [31]. The 2A peptides are from picornavirus and are extremely rare in the mammalian genome. The “cleavage” caused by the 2A peptide is not proteolytic; instead it promotes ribosomal skipping by preventing the normal peptide bond formation between glycine and proline (Figure 2A) [32]. The 2A peptide-mediated cleavage does not affect the translation efficiency of the downstream coding sequences. mCherry serves as a marker of expression and thus as an internal reference protein. Two distinct 2A peptide linkers were used. The E2A linker (from equine rhinitis virus) was inserted between NYFP-MEIOB and CYFP-SPATA22, while the F2A linker (from foot-and-mouth disease virus) was engineered between CYFP-SPATA22 and mCherry (Figure 2B). HEK293 cells were transfected with this multi-protein expression construct. Both green fluorescence (restored by MEIOB–SPATA22 interaction) and red fluorescence (mCherry) were observed in transfected cells (Figure 2C). As a negative control, the interaction-disrupting point mutation (D383A) in MEIOB was introduced in the multi-protein expression vector (Figure 2B). As expected, cells transfected with this mutant vector were positive for mCherry but negative for green fluorescence (YFP) (Figure 2C). These results showed that the BiFC assay for the MEIOB–SPATA22 interaction was robust.

We next cloned the NYFP-MEIOB CYFP-SPATA22 mCherry insert from the BiFC vector (Figure 2A) into the pcDNA5/FRT/TO vector (tetracycline-inducible). A stable cell line (No. 20) was generated by transfection of the Flp-In T-REx 293 cells (Thermo Fisher) with the pcDNA5-BiFC (wild type) vector and the Flp recombinase-expressing plasmid [33]. The advantage of a stable Tet-inducible BiFC cell line is threefold: (1) inducible expression, (2) no need for transfection, and (3) increased reproducibility.

### High-throughput screening of compound libraries

We screened a total of 47 800 compounds from four libraries (MicroSource purified natural product library, SelleckChem bioactive library, ChemDiv’s SMART library, and ChemBridge’s Core set) using the BiFC stable cell line in the 384-well format. As reconstitution of the YFP fluorophore in the BiFC system is irreversible [28], inhibitors must be added prior to the appearance of fluorescence. YFP fluorescence usually appears 8–12 h after transfection [29], due to the time required for expression, protein–protein binding, YFP complementation, and fluorophore maturation [28]. In our screen, compounds and tetracycline were added simultaneously to the cells. After 24 h, cells were fixed for imaging. In the pilot screen, we screened 800 purified natural products (MicroSource) and obtained 6 compound hits defined by 3 standard deviations from the aggregate mean of the YFP/mCherry ratio in all wells and more than 50% of cell viability (Supplementary Table S1). Isocotoin was the most potent compound identified with 60% inhibition of the YFP fluorescence. In the second pilot screen, we screened a library of 3000 small molecules (SelleckChem) with annotated biological activities and obtained three hits (Supplementary Table S2).

We next screened 44 000 compounds from ChemDiv’s SMART library and ChemBridge’s core set. Because there were no known inhibitors of the MEIOB–SPATA22 interaction, isocotoin identified in the pilot screen was used as a positive control in these large-scale screens. Compounds were added to a final concentration of 20 μM. We determined candidate hits by the following criteria: (a) the YFP/RFP ratio z-score was less than −3 (three standard deviations); (b) cell viability (total cell POC) was > 80%; (c) RFP integrated intensity z-score was between −3 and 3 (excluding toxicity and autofluorescence). By these criteria, a total of 323 compounds were identified at a hit rate of 0.7%: 143 from ChemDiv’s SMART library (Supplementary Table S3) and 180 from ChemBridge’s core set (Supplementary Table S4).

### Isocotoin inhibits the MEIOB–SPATA22 interaction

**Figure 3:**
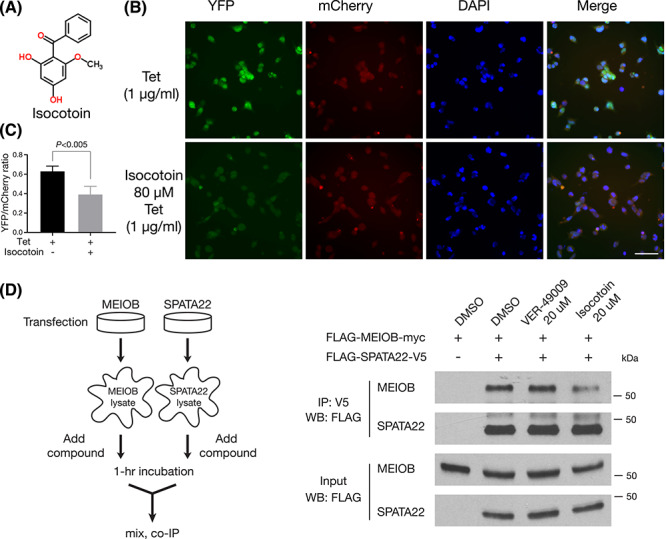
Isocotoin inhibits the MEIOB–SPATA22 interaction. (A) Chemical structure of isocotoin. (B) Isocotoin reduces the YFP fluorescence in the BiFC stable cell lines. The MEIOB–SPATA22 BiFC stable cells (No. 20) were treated for 24 h with tetracycline (1 μg/ml) alone or both isocotoin (80 μM) and tetracycline. Scale bar, 50 μm. (C) Quantification of YFP/mCherry fluorescence (ratio on the y-axis). Four images of each treatment (as in panel B) were quantified with ImageJ. ~ 1000 cells in each treatment were quantified. (D) Co-immunoprecipitation analysis with and without compounds. The experiment flowchart is shown on the left. HEK293 cells were transfected separately with MEIOB- and SPATA22-expressing plasmids. Epitope-tagged constructs were previously described [20]. The final concentration of compounds in the lysate was 20 μM. VER-49009 is an inhibitor of HSP90 [35].

Isocotoin from the natural product library (C_14_H_12_O_4_; CAS# 81525-12-4; MW, 244; Figure 3A) reduced the YFP fluorescence in tetracycline-treated BiFC cells (Supplementary Table S1). We retested the effect of isocotoin with resynthesized compound. In the BiFC cells, isocotoin (80 μM) sharply reduced the YFP fluorescence but had no effect on the mCherry red fluorescence (Figure 3B). Isocotoin (80 uM) reduced the YFP/mCherry ratio by 37% (Figure 3C). To test whether isocotoin inhibits the MEIOB–SPATA22 interaction, we performed co-immunoprecipitation with and without isocotoin (Figure 3D). HEK293 cells were transfected separately with MEIOB and SPATA22. Protein lysates were incubated with DMSO, VER-49009, or isocotoin, prior to mixing and co-immunoprecipitation. VER-49009 was a hit compound from the SelleckChem library screening (Supplementary Table S2). VER-49009 is a potent inhibitor of HSP90, a molecular chaperone involved in stress responses, folding, and stability of client proteins [34, 35]. Inhibition of HSP90 causes degradation of substrate proteins through the ubiquitin–proteasome pathway [36]. Much less MEIOB was co-immunoprecipitated with SPATA22 in the presence of isocotoin than the DMSO control and VER-49009, showing that isocotoin inhibits the MEIOB–SPATA22 complex formation but VER-49009 does not (Figure 3D).

### Isocotoin promotes the degradation of MEIOB and SPATA22

**Figure 4:**
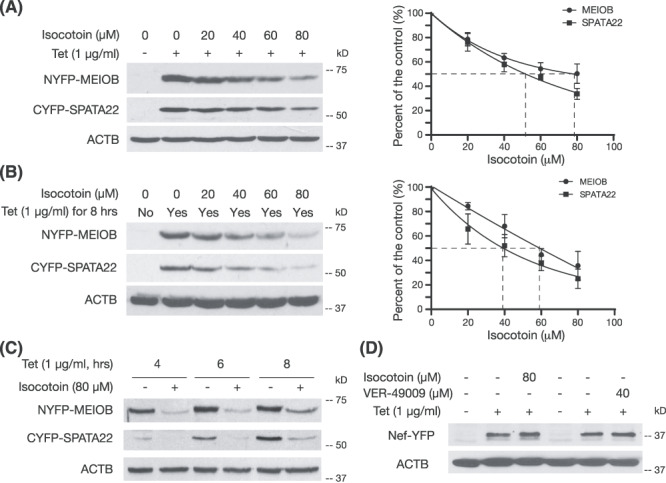
Isocotoin promotes degradation of MEIOB and SPATA22. (A) Dose–response curve of isocotoin on the degradation of MEIOB and SPATA22. The BiFC stable cells were treated simultaneously with tetracycline (1 μg/ml) and various concentrations of isocotoin (0–80 μM) for 24 h. Note that transcription of *Meiob* and *Spata22* was continuous, due to the presence of tetracycline in the medium in the 24-h duration of experiments. The experiments were performed three times. Band intensity was quantified using ImageJ and plotted on the right. (B) Dose–response curve of isocotoin on the degradation of existing MEIOB and SPATA22. The BiFC stable cells were treated with tetracycline (1 μg/ml) for 8 h, washed, and cultured in fresh medium with isocotoin only (0–80 μM) for 16 h. The experiments were performed three times and plotted on the right. (C) Isocotoin promotes degradation of MEIOB/SPATA22 in short-term treatments. The BiFC stable cells were treated with tetracycline (1 μg/ml) alone or simultaneously with tetracycline (1 μg/ml) and isocotoin (80 μM) for 4, 6, and 8 h. Tet: tetracycline. (D) Lack of effect of isocotoin on degradation of Nef-YFP. The Nef-NYFP-Nef-CYFP BiFC stable cells were treated for 8 h and then collected for Western blot analysis. Note that VER-49009 lacks effect on the stability of Nef-YFP.

We examined the protein abundance in the BiFC cells treated with tetracycline and isocotoin simultaneously (Figure 4A). Without tetracycline, MEIOB and SPATA22 were not expressed. Isocotoin reduced the abundance of both MEIOB and SPATA22 in a dose-dependent manner. The isocotoin IC50 was 75 ± 10 μM for MEIOB and 51 ± 8 μM for SPATA22. In this experiment, transcription was continuously induced by tetracycline (Figure 4A). We next performed a pulse–chase experiment (Figure 4B). The BiFC cells were treated with tetracycline for 8 h, washed, and subsequently treated with isocotoin for 16 h. Consistently, isocotoin decreased the abundance of MEIOB and SPATA22 in a dose-dependent manner, suggesting that isocotoin affects the protein stability after complex formation. As expected, the isocotoin IC50 was lower: 62 ± 6 μM for MEIOB and 37 ± 11 μM for SPATA22 (Figure 4B). Furthermore, isocotoin promoted degradation of MEIOB and SPATA22 in short-term treatments (4–8 h) (Figure 4C). HIV Nef protein forms homodimers [29]. Isocotoin did not promote degradation of the Nef-YFP protein in the Nef-YFP BiFC cells, suggesting that isocotoin does not inhibit YFP (Figure 4D). These results demonstrated that isocotoin causes degradation of MEIOB and SPATA22.

## Discussion

We have demonstrated that the conserved D383 residue in MEIOB is essential for its function in meiosis in vivo. Importantly, both MEIOB and SPATA22 proteins were absent in the *Meiob*^D383A/D383A^ testes (Figure 1C), showing that disruption of their interaction destabilizes both proteins in vivo. Therefore, small molecule inhibitors of their interaction would lead to their degradation and thus infertility. Because MEIOB and SPATA22 are only expressed in meiotic germ cells, specific inhibition of their interaction will probably not affect somatic cells [12, 13]. In addition, since MEIOB and SPATA22 are not expressed in spermatogonial stem cells, the contraceptive effect of these inhibitors would be reversible in one cycle of spermatogenesis. As genetic and molecular studies indicate that these two proteins can be manipulated to result in a “pure sterile” phenotype, these two proteins constitute novel validated male contraceptive targets.

We have developed a cell-based BiFC assay for screening inhibitors of the MEIOB–SPATA22 interaction. The BiFC assay has been developed for investigating protein–protein interactions or protein dimerization and for chemical library screening [28, 29, 37]. In our BiFC assay, a single tricistronic vector has the advantage of expressing all three proteins in the same cell (Figure 2B). However, a single vector still requires transfection. Even though transfection efficiency in HEK293 cells is usually very high, protein expression still varies from cell to cell. We have generated a MEIOB–SPATA22 BiFC HEK293 stable cell line, in which transcription is tetracycline-inducible. The use of an inducible stable cell line significantly simplified the screening process and increased uniformity of protein expression levels. This BiFC stable cell line can be readily used for screening of more compounds in the future. Because this assay is cell-based, cytotoxic compounds can be excluded. In addition, autofluorescent compounds can also be excluded. Another advantage of this assay is that compounds identified are expected to be cell-permeable.

High-content screening of chemical libraries using our BiFC assay has identified compound hits for further study (Supplementary Tables S1–S4). Isocotoin was one of the most potent compounds identified in this screen. Isocotoin causes degradation of MEIOB and SPATA22 in the BiFC cell line (Figure 4). Isocotoin inhibits the complex formation of MEIOB and SPAT22 (Figure 3D). Intriguingly, isocotoin also promotes degradation of MEIOB and SPATA22 even after they form complexes (Figure 4B). One possible explanation is that isocotoin not only inhibits the MEIOB–SPATA22 interaction but also promotes their degradation through an unknown mechanism even after they form a complex. The biochemical basis of action by isocotoin in the degradation of MEIOB and SPATA22 warrants further investigation. This study shows that the BiFC assay can be used for screening not only inhibitors of protein–protein interaction but also compounds that promote protein degradation.

## Authors’ contributions

YX, RL, and LZ carried out the experiments. NAL performed microinjection of zygotes. II and DCS performed the compound screen. YX, RL, II, and DCS analyzed data. PJW, YX, II, and DCS wrote the manuscript. All authors commented on the manuscript.

## Supplementary Material

Figure_S1_ioaa062

Table_S1_4_ioaa062
