# Supplementary figures and images for: A cell-based high-content screen identifies isocotoin as a small molecule inhibitor of the meiosis-specific MEIOB–SPATA22 complex
Source: Biol Reprod. 2020 Apr 25;103(2):333–42. doi: 10.1093/biolre/ioaa062 (PMC7523692; doi:10.1093/biolre/ioaa062)

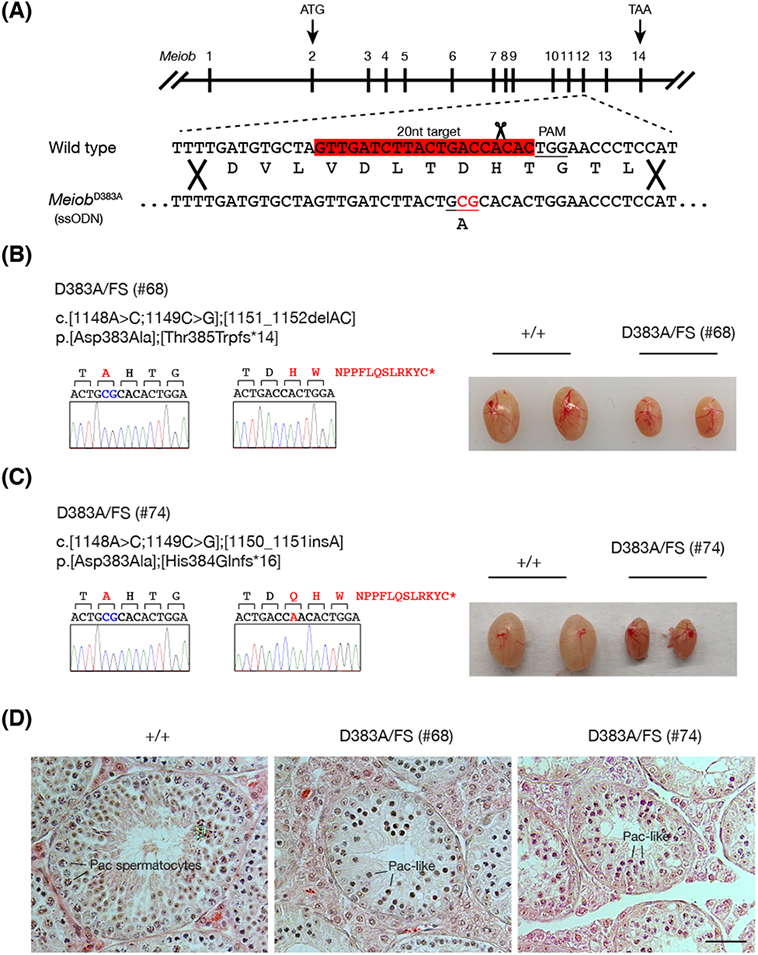

Supplement: Figure_S1_ioaa062 [file figure_s1_ioaa062.png]
